# A165 FCGBP IN THE COLON IS SPATIALLY DEPENDENT ON MUC2 EXPRESSION AND IS DEGRADED IN DEXTRAN SULFATE SODIUM-INDUCED COLITIS

**DOI:** 10.1093/jcag/gwac036.165

**Published:** 2023-03-07

**Authors:** A Kim, F Moreau, K Chadee

**Affiliations:** Microbiology, Immunology, and Infectious Diseases, University of Calgary, Calgary, Canada

## Abstract

**Background:**

The colonic mucus bilayer is an integral innate host defense mechanism that provides a physical barrier separating the lumen and its contents from the underlying epithelium. This essential barrier is produced by specialized secretory goblet cells of which Muc2 mucin is its primary product. IgG-Fc-binding protein (Fcgbp) is the second most abundant protein produced by goblet cells, which has a suggested function of crosslinking with Muc2 to stabilize the structural integrity of mucus. FCGBP is observed to decrease preceding the onset of inflammation in ulcerative colitis patients, leading to spatially distinct structural mucus weakening to contribute to the pathogenesis of the disease.

**Purpose:**

Fcgbp is altered regionally in the gut and plays a role in the pathogenesis of dextran sulfate sodium (DSS)-induced colitis. The specific aims are:

To characterize the spatial expression of Muc2 and Fcgbp basally in goblet cells

To quantify alterations in Muc2 and Fcgbp in response to DSS-induced colitis and at restitution of disease

**Method:**

mRNA and protein expression in *Muc2*^*+/+*^ and *Muc2*^*-/*-^ C57BL/6 littermates were analyzed by RT-qPCR and Western blotting, respectively. Mucin granules were isolated from colonic goblet cells and the proteome quantified by liquid chromatography and tandem mass spectrometry (LC-MS/MS). Colitis was induced in *Muc2*^*+/+*^ mice with 3.5% (w/v) DSS in tap water for five days, whereas *Muc2*^*-/*-^ littermates were given 1.5% (w/v) DSS in tap water for three days, ad libitum. Mice were given regular tap water for the remainder of the experiment to allow restitution of inflammation. Disease activity index (DAI) was scored based on weight loss. Mice were sacrificed at various time points up to 10 days, and colons excised and sectioned for histopathology analysis.

**Result(s):**

LC-MS/MS of mucin granules run under reducing and non-reducing conditions confirmed that Muc2 and Fcgbp were the most abundant proteins in mucin granules and were non-covalently bound to each other. mRNA and protein expression of Muc2 and Fcgbp were highly expressed in the mid colon, and regulation of Fcgbp was unaffected in *Muc2*^*-/*-^ littermates. In response to DSS-induced colitis in *Muc2*^*+/+*^ mice, Muc2 transcription rapidly increased in all regions of the colon with highest expression in the mid colon. In contrast, Fcgbp transcription increased in the mid and distal colons and peaked during highest disease activity. Interestingly, Fcgbp protein expression was abrogated in the mid colon even at restitution. In *Muc2*^*-/*-^ mice, Fcgbp transcription decreased during disease onset but returned to normal levels following removal of DSS.

**Image:**

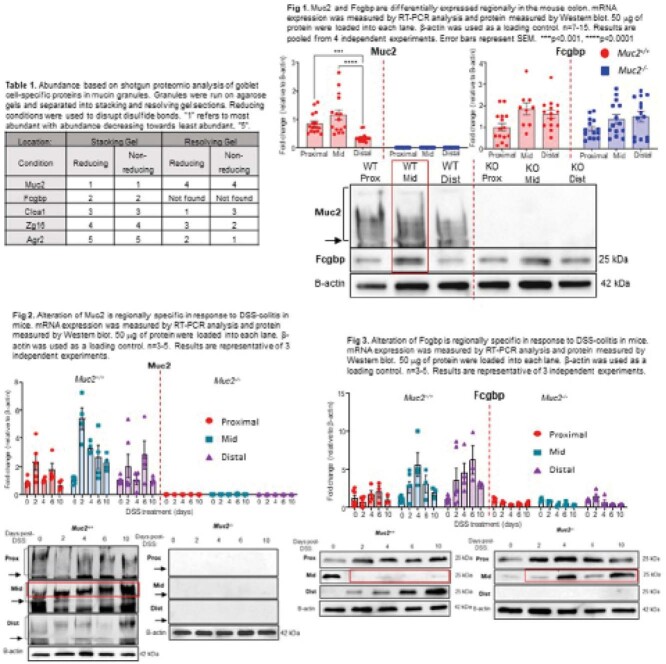

**Conclusion(s):**

This study demonstrates that in response to DSS, Fcgbp expression was spatially degraded at the onset of disease and remained low at restitution. The disappearance of Fcgbp in the mid colon of *Muc2*^*+/+*^ littermates, despite Muc2 restoration, suggests that the mucus barrier remains structurally altered and functionally impaired at restitution. Supported by CIHR

**Disclosure of Interest:**

None Declared

